# Endothelial cells regulate neural crest and second heart field morphogenesis

**DOI:** 10.1242/bio.20148078

**Published:** 2014-07-04

**Authors:** Michal Milgrom-Hoffman, Inbal Michailovici, Napoleone Ferrara, Elazar Zelzer, Eldad Tzahor

**Affiliations:** 1 Department of Biological Regulation, Weizmann Institute of Science, Rehovot 76100, Israel; 2 Department of Pathology, University of California at San Diego, La Jolla, CA 92093, USA; 3 Department of Molecular Genetics, Weizmann Institute of Science, Rehovot 76100, Israel

**Keywords:** Endothelial cell, Neural crest, Second heart field, ECM

## Abstract

Cardiac and craniofacial developmental programs are intricately linked during early embryogenesis, which is also reflected by a high frequency of birth defects affecting both regions. The molecular nature of the crosstalk between mesoderm and neural crest progenitors and the involvement of endothelial cells within the cardio–craniofacial field are largely unclear. Here we show in the mouse that genetic ablation of vascular endothelial growth factor receptor 2 (Flk1) in the mesoderm results in early embryonic lethality, severe deformation of the cardio–craniofacial field, lack of endothelial cells and a poorly formed vascular system. We provide evidence that endothelial cells are required for migration and survival of cranial neural crest cells and consequently for the deployment of second heart field progenitors into the cardiac outflow tract. Insights into the molecular mechanisms reveal marked reduction in Transforming growth factor beta 1 (Tgfb1) along with changes in the extracellular matrix (ECM) composition. Our collective findings in both mouse and avian models suggest that endothelial cells coordinate cardio–craniofacial morphogenesis, in part via a conserved signaling circuit regulating ECM remodeling by Tgfb1.

## INTRODUCTION

The anterior region of the embryo gives rise to the head and heart through overlapping morphogenetic processes. Pharyngeal mesoderm cells contribute to significant parts of the developing heart and the head musculature (Grifone and Kelly, 2007; Tzahor, 2009; Tzahor and Evans, 2011), as well as to endothelial cells (EC). Pharyngeal mesoderm surrounds the pharynx and contains both paraxial and splanchnic mesoderm regions that later form the core of the pharyngeal arches (Nathan et al., 2008). In addition to pharyngeal muscles, pharyngeal mesoderm also contributes to both poles of the heart, following the formation of the linear heart tube. The cardiogenic population of pharyngeal mesoderm cells is known as the second heart field (SHF), or anterior heart field (Buckingham et al., 2005; Tzahor and Evans, 2011). Perturbations in the recruitment of these cells to the heart tube can lead to a wide range of congenital heart defects. Such defects occur in nearly 1% of live births, reflecting the complex cellular processes underlying heart development (Srivastava, 1999; Buckingham et al., 2005).

From an embryonic point of view, the development of the head–heart region should be considered as a single morphogenetic field, in which every tissue in it is influenced by neighboring tissues (Hutson and Kirby, 2003). Due to the anatomical proximity during early embryogenesis and overlapping progenitor populations, cardiac and craniofacial birth defects are often linked (Grifone and Kelly, 2007; Tzahor, 2009; Tzahor and Evans, 2011). DiGeorge syndrome (DGS) is one of the most frequent chromosomal deletion syndrome in humans (Yamagishi and Srivastava, 2003; Baldini, 2005). Its clinical features broadly include cardiac defects, craniofacial and aortic arch anomalies. Previous studies addressing DGS etiology implicate a series signaling pathways such as FGF (Guo et al., 2011), VEGF (Stalmans et al., 2003), retinoic acid (Roberts et al., 2006; Ryckebüsch et al., 2010) and TGFbeta (Wurdak et al., 2005; Choudhary et al., 2006). These studies highlight the linkage in signaling circuits in different cell types during cardiac and craniofacial development processes, whose nature are largely unknown.

Cranial neural crest (NC) cells migrate to the pharyngeal arches (Noden, 1983; Trainor and Tam, 1995). Craniofacial malformations are attributed to defects in the patterning, proliferation, migration or differentiation of this cell population (Noden and Trainor, 2005). Cardiac NC located caudal to the cranial NC territory, were found to be critical for normal heart development (Kirby et al., 1983). Malfunction of the cardiac NC affects the caudal cardio–craniofacial field whereas the involvement of cranial NC population in cardiac development is far less studied.

Blocking agents against various ECM molecules perturb NC cell migration (Tucker, 2004). The ECM is known to regulate numerous cellular processes as are ECM modulating factors, which affect the geometry and composition of the ECM. Individual ECM components are laid down, cross-linked, and organized together via covalent and noncovalent modifications. Deregulation of these control mechanisms can lead to various human pathologies. TGFβ signaling has been shown to be an important modulator of the ECM by stimulating the synthesis of ECM components (Wells and Discher, 2008). Conventional knockout of either Tgfbr2 (Oshima et al., 1996) or Tgfb1 (Dickson et al., 1995) in mice results in developmental retardation and early mortality attributed to the loss of EC integrity. Indeed, Tgfb1 signaling plays a dominant role in development the vascular network (ten Dijke and Arthur, 2007).

During vertebrate embryogenesis the circulatory system is the first functioning physiological system to emerge together with the blood system. Its main function is to deliver oxygen and nutrients to the developing tissues, although accumulating evidence supports a perfusion-independent signaling role(s) for EC. Development of the liver and pancreas has been shown to be dependent on signals from blood vessels (Lammert et al., 2001; Matsumoto et al., 2001). Thus, EC can provide instructive regulatory signals to other cell types (Cleaver and Melton, 2003).

In this study we addressed the roles of EC in the morphogenesis of the cardio–craniofacial field. Embryos deficient of EC, via the ablation of Flk1 in the cardio–craniofacial mesoderm, exhibited arrested development of NC and SHF progenitors in addition to vascular defects. Several ECM genes were affected in these conditional mutants, including EC-derived Tgfb1, suggesting that EC are required for the integrity of the ECM. Conditional TgfbR2 knockout in mesoderm progenitors resulted in cardiac SHF phenotype supporting a critical role of Tgfb1 signaling in the crosstalk within the cardio–craniofacial field. Finally, we were able to phenocopy the mouse phenotype in the chick model using a VEGFR2 inhibitor. Chick embryos treated with the inhibitor had similar phenotypic and molecular changes as in the mouse model. Taken together, our findings suggest that Tgfb1 secreted by EC regulates ECM remodeling that is crucial for proper cardio–craniofacial morphogenetic development.

## MATERIALS AND METHODS

### Preparation of chick/quail embryos

Fertilized chick/quail eggs were incubated at 38°C under 80% humidity; embryos were staged according to Hamburger and Hamilton.

### Inhibitors administration

The VEGFR tyrosine kinase inhibitor II (EMD Millipore) at 5 mg/ml in DMSO was diluted 1:150,000 in PBS and administered to stage 5 Quail/chick embryos. As a control, the same dilution of DMSO was used. Embryos were then further incubated until reaching the required developmental stage.

### RNA extraction and amplification

RNA was harvested using a microRneasy Kit (Quaigen). cDNA was synthesized from DNase-treated total RNA, using a M-MLV reverse transcriptase-mediated extension of random primers, followed by a RT-PCR amplification using different sets of primers (available upon request).

### In situ hybridization

Whole-mount in situ hybridization was performed using digoxigenin-labeled antisense riboprobes synthesized from the cDNAs. A full list of the in situ hybridization probes and a detailed protocol are available upon request. Images were obtained using a Leica MZ16FA stereomicroscope attached to a digital camera (DC300F, Leica Microsystems).

### Mouse lines

Conditional knockout embryos were generated using either *MesP1Cre*, or *Tie2Cre* mice, crossed with the TgfbR2 conditional mice (Chytil et al., 2002; Koni et al., 2001; Saga et al., 1996). For FACS isolation *Tie2Cre* crossed with the *Rosa26YFP* reporter or *Pax3Cre* crossed with the *Rosa26Tomato* reporter.

All animal experiments were performed in accordance with the Weizmann Institute of Science regulations for animal care and handling.

### Immunofluorescence

Cryo sections were blocked with 5% horse serum, and incubated with the following antibodies: QH1, PECAM1, ISL1, PAX7 (DSHB), AP-2 alpha (Novus Biologicals), P-smad2/3 and NKX2.5 (Santa-Cruz), Col1 and Tgfb1 (Abcam) and FLK1 supplied by Philip Thorpe's lab (UT Southwestern Medical Center). Secondary antibodies used were Cy2-, Cy3-, or Cy5-conjugated anti-mouse, anti-rat or anti-rabbit IgG, Cy3-conjugated anti-mouse IgG1, and Cy5-conjugated anti-mouse IgG2b (1:100, Jackson ImmunoResearch).

## RESULTS

### Flk1 and endothelial cells regulate cardiac and craniofacial morphogenesis

**Fig. 1.**
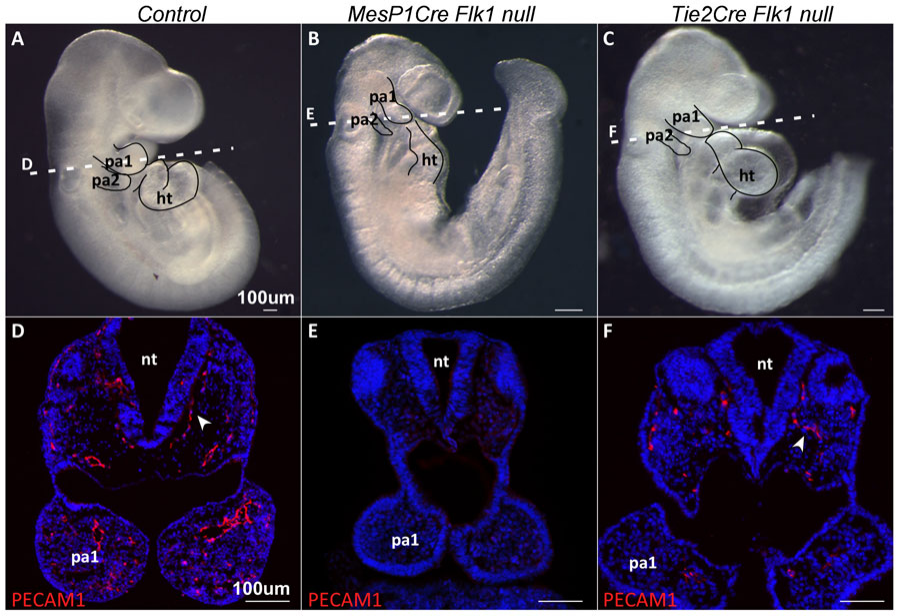
Genetic ablation of *Flk1* in mouse embryos leads to a loss of endothelial cells. (A) E9.5 control embryo compared to matching stage (B) *Mesp1Cre Flk1* and (C) *Tie2Cre Flk1* mutants. Note the poorly developed heart and pharyngeal arches in mutant embryos. Dotted lines indicate the level of sections. Transverse sections of E9.5 (D) control embryo, (E) *Mesp1Cre Flk1* and (F) *Tie2Cre Flk1* mutants. Sections are stained with the endothelial marker PECAM1 showing decrease in endothelial cells in mutant embryos. Scale bars: 100 µm.

The expression of Vascular Endothelial Growth Factor Receptor 2 (Flk1) in early mesodermal cells marks progenitors with a broad lineage potential, although it is thought that this gene is primarily necessary for the formation of endothelial and hematopoietic lineages (Shalaby et al., 1995; Motoike et al., 2003). We have previously used the conditional allele of *Flk1* with several mesodermal Cre deletors, as a specific and effective method to induce EC dysfunction (Milgrom-Hoffman et al., 2011). Conditional ablation of this gene in the MesP1 lineage, encompassing the entire anterior mesoderm of the embryo (Saga et al., 1999), resulted in loss of EC in the anterior region of the embryo and mutant embryos die at E9.5 (Fig. 1A,B,D,E). These mutant embryos were developmentally retarded; the pharyngeal arches were smaller and malformed compared to control embryos, the heart tube was shorter and not properly looped (Fig. 1B,E).

We next generated EC specific *Flk1* cKO mutants using the *Tie2Cre*. As with the *MesP1Cre*, we observed a reduction in PECAM1^+^ EC (Fig. 1F). The phenotype of the *Tie2Cre Flk1* mutant embryos was milder compared to the *Mesp1Cre Flk1* mutants, yet preserved the trend of hypomorphic pharyngeal arches and abnormal heart looping (Fig. 1C,F). Results for *Tie2Cre Flk1* cKO mutants are mostly shown in supplementary material Figs S1 and S2, and *Flk1 cKO* mutants refer to *MesP1Cre Flk1* mutants. Taken together, our *Flk1* cKO mutants reveal a possible link between the loss of EC and a cardio–craniofacial phenotype. These findings suggest a regulatory role for EC in the morphogenesis of the cardio–craniofacial field.

### Differential effects of endothelial cells on cranial neural crest and pharyngeal mesoderm progenitors

**Fig. 2.**
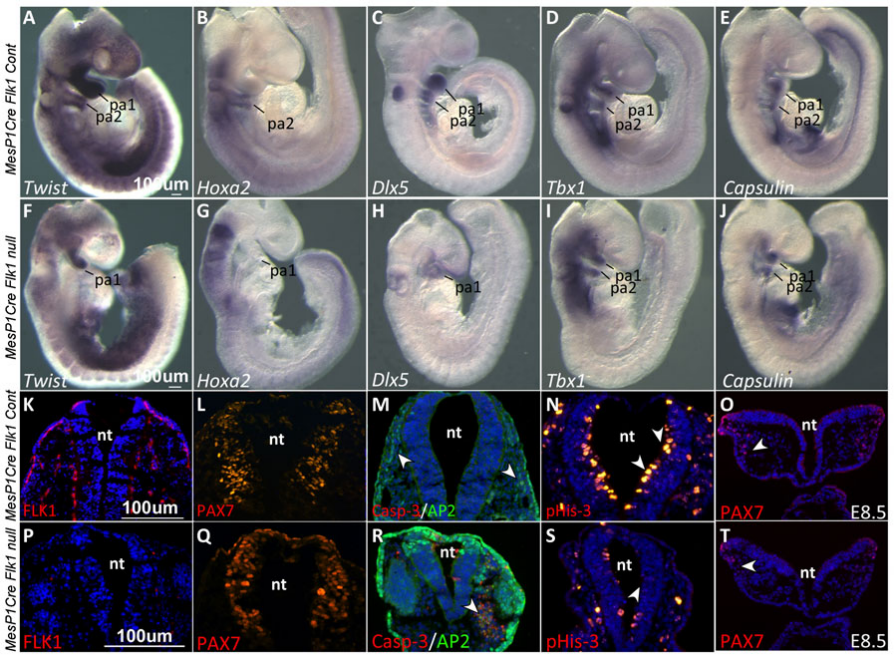
Cranial neural crest defects in *Flk1* cKO mutants. In situ hybridization in E9.5 control and mutant embryos for the NC cell's markers (A,F) *Twist*, (B,G) *HoxA2* and (C,H) *Dlx5*. Note the down-regulation of these genes in the mutant embryo PAs. In situ hybridization for the mesodermal markers (D,I) *Tbx1* and (E,J) *Capsulin*. (K–T) Immunostaining on transverse sections of E9.5 embryos for (K,P) the endothelial marker FLK1 and (L,Q) the NC progenitor marker PAX7. (M,R) Co-staining for the NC marker AP2 and cell death marker Casp-3. (N,S) Staining for pHis3 and comparison of neuroepithelium proliferation in control and mutant embryos, indicated by arrowheads. (O,T) E8.5 embryo sections stained for the NC progenitor marker PAX7. All fluorescent images except panels L and Q are counterstained with DAPI (blue). pa, pharyngeal arch; nt, neural tube. Scale bars: 100 µm.

In order to gain insights into the craniofacial phenotype observed in *MesP1Cre* and *Tie2Cre Flk1* cKO mutants, we performed in situ hybridization for different cranial NC and mesoderm markers, the two resident cell populations in the pharyngeal arches. Strikingly, NC markers were completely absent from the second arch of the mutant embryos (Fig. 2; supplementary material Fig. S1). *Twist* expression was decreased in the first pharyngeal arch of *Flk1* cKO mutants compared to the control embryos, and undetected in the second arch (Fig. 2A,F). *HoxA2*, a specific marker for second arch NC cells (Kanzler et al., 1998), was undetected in the *Flk1* cKO mutants (Fig. 2B,G). Likewise, *Dlx5* was downregulated in the first arch and undetected in the second arch of mutant embryos compared to the control (Fig. 2C,H).

In comparison to this dramatic effect on NC gene expression, the mesoderm markers *Tbx1* and *Tcf21* (*Capsulin*) were expressed at normal levels in the core of the two pharyngeal arches in *MesP1Cre Flk1* mutants as well as in control embryos (Fig. 2D,E,I,J,). In conclusion, these results indicate that ablation of EC in the *Flk1* cKO mutants specifically affect NC but not the pharyngeal mesoderm gene expression.

We next analyzed NC specific markers at a higher resolution by staining E9.5 embryonic sections at the level of the second arch. While FLK1 expressing cells were detected in control embryos, underlining the ectoderm and the neuroepithelium, its expression in *Flk1* cKO mutants was undetectable (Fig. 2K,P). PAX7 staining was comparable in the control and mutant embryos (Fig. 2L,Q) indicating that the specification of NC within the neural tube was unaffected. Cranial NC cell death was observed by co-staining for AP2 and Caspase-3 (Fig. 2M,R). Apart from the specific NC phenotype, sectioning the embryos enabled us to identify an additional neuroepithelium phenotype in the *Flk1* cKO mutants, which had a thin and deformed neural tube, accompanied by a marked decrease in the proliferation of the neuroepithelium (Fig. 2N,S). Notably, the NC phenotype was also evident at E8.5 where PAX7^+^ NC cells in the mutant migrated to a lesser extent compared to the control (Fig. 2O,T). Taken together, we suggest that EC loss affects specifically NC migration and survival but not its specification.

**Fig. 3.**
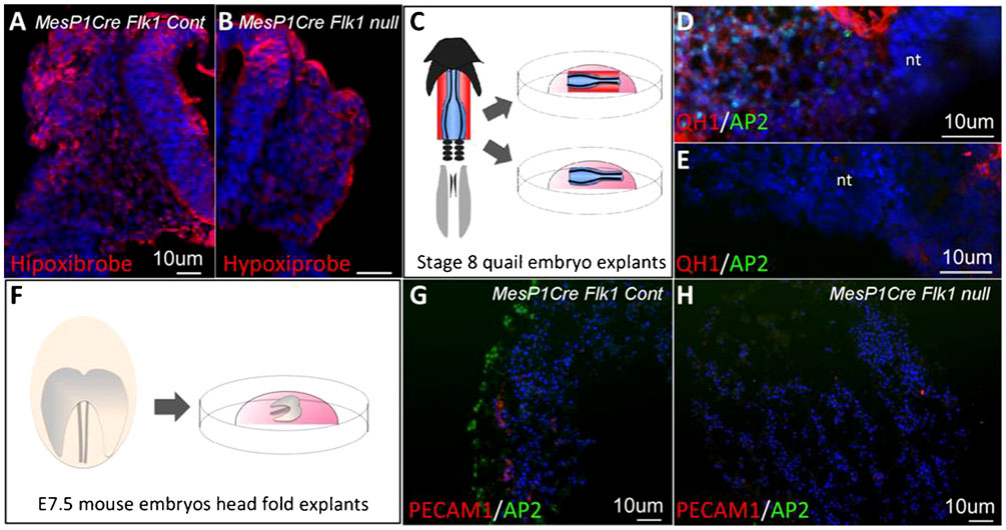
Hypoxia-independent functions of EC on NC development. (A,B) Transverse sections of E9.5 *Mesp1Cre Flk1* control and mutant embryos stained with hypoxiprobe. (C) Cranial neural tubes (blue) of stage 8 quail embryos dissected either with or without a small portion of the underlying mesoderm (red). Explants were grown for 48 hours in culture. Staining for the endothelial (QH1) and NC (AP2) markers. (D) Cultures of neural tube explants with the underlying mesoderm encompassing endothelial cells (n = 3/3) or (E) neural tube explant alone (n = 3/3). (F) The head region of E7.5 *Mesp1Cre Flk1* control and mutant embryos dissected and cultured for 48 hours. Co-staining for PECAM1 and AP2 in (G) control embryo explants (n = 5/5) and (H) mutant embryo explants (n = 2/2). Fluorescent images are counterstained with DAPI (blue). Scale bars: 10 µm.

In order to exclude the possibility that the cardio–craniofacial defects were not a consequence of poor vasculature and impending oxygen delivery we compared the hypoxic levels between control E9.5 and *Flk1* cKO embryos. Both control and mutant embryos were positive for Hypoxiprobe staining within the neural tube and surrounding tissues in a comparable manner (Fig. 3A,B). Next, we employed explant culture assays to determine the effect of EC on cranial NC migration and survival, by eliminating circulation and hypoxia as influencing factors (Fig. 3C–H). Neural crest explants composed of the dorsal neural tube from stage 8 quail embryos were dissected with or without the adjacent mesoderm (Fig. 3C) and cultured for 24 hours followed by staining for NC and endothelial markers (Fig. 3D,E). AP2 staining was undetected in explants lacking the adjacent mesoderm, which includes QH1^+^ EC. Next we used mouse embryo explants of the head region of E7.5 control and *Flk1* cKO mutant embryos (Fig. 3F). Explants were then sectioned and stained for NC and endothelial markers (Fig. 3G,H). As in the avian model, the lack of EC in the *Flk1* mutants was associated with a failure of NC migration. Combined with the differential effects on pharyngeal mesoderm and NC gene expression patterns (Fig. 2), our data do not support the classical explanation of a poorly formed vascular system to be the main cause for the loss of the cranial NC, but rather a signaling mechanism between EC and NC populations.

### Endothelial cells affect the formation of second heart field derived structures

**Fig. 4.**
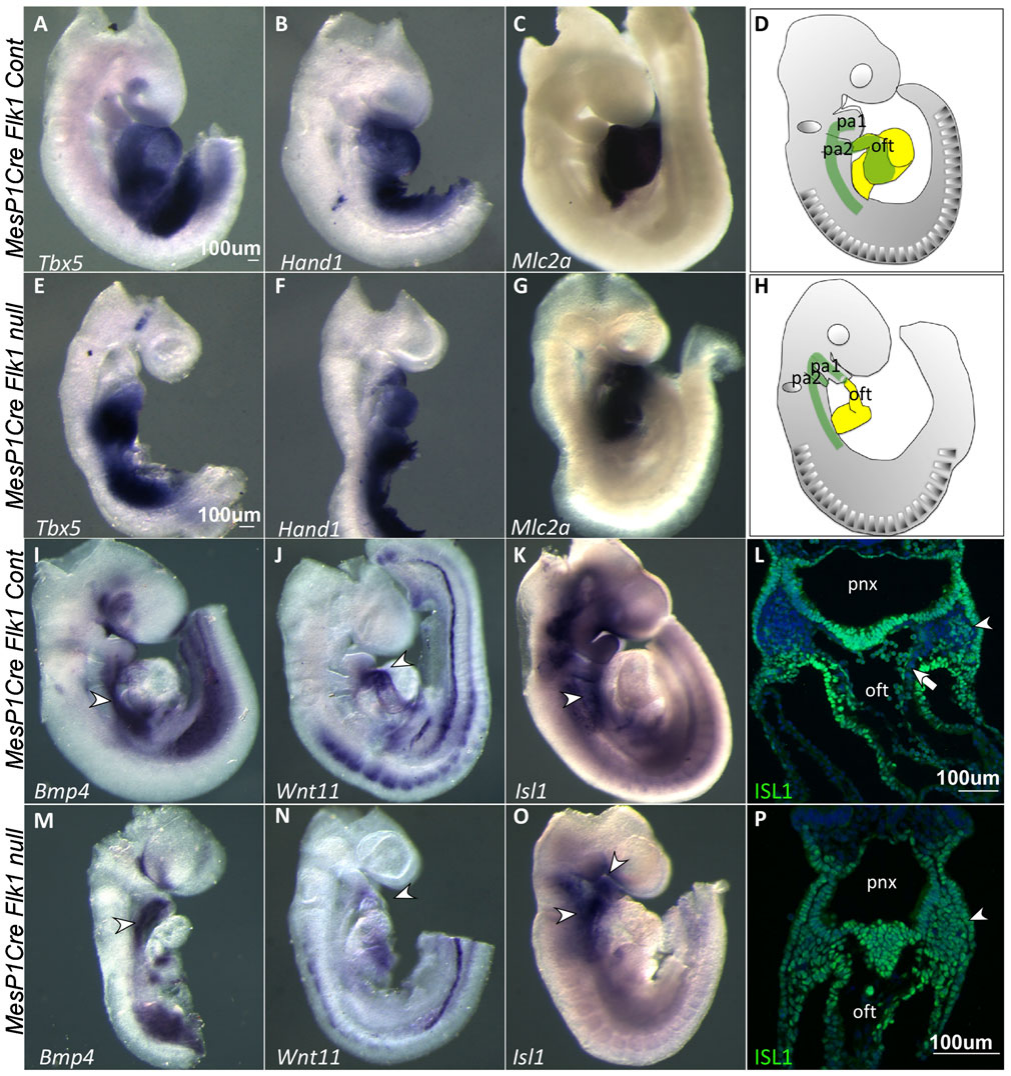
Second heart field phenotype in *Flk1* cKO mutants. In situ hybridization at E9.5 for first and second heart field markers in control and mutant embryos. FHF markers (A,E) *Tbx5*, (B,F) *Hand1* and (C,G) *Mlc2a* expression were comparable between control and mutant embryos. (D,H) A schematic representation of the spatial location of second (green) and first (yellow) heart fields in control and *Mesp1Cre Flk1* mutant embryos. SHF markers (I,M) *Bmp4*, (J,N) *Wnt11* and (K,O) *Isl1* were affected in the mutant embryos as indicated by arrowheads. (L,P) Immunostaining for ISL1 on transverse sections of control and *Mesp1Cre Flk1* mutant embryos as indicated by arrowheads. Arrow indicates NC cells entering the OFT. Counterstaining for nuclei with DAPI (blue). pa, pharyngeal arch; nt, neural tube; pnx, pharynx; oft, outflow tract. Scale bars: 100 µm.

Our data revealed that NC development was compromised in *Flk1* cKO mutants (in both *MesP1* and *Tie2* lineages, Fig. 2). We next asked whether the effect on NC development can be linked to the observed cardiac defects, which include hypotrophy and aberrant heart tube looping (Fig. 1). Expression of the first heart field markers *Tbx5*, *Hand1* and *Mlc2a* (Klaus et al., 2007) was comparable between *Flk1* cKO mutant and control embryos (Fig. 4A–C,E–G). In contrast, the expression of SHF markers was changed. *Wnt11*, which is expressed in the outflow tract (OFT), was completely missing in mutant embryos (Fig. 4J,N; supplementary material Fig. S2), indicating a shortening of the OFT in *Flk1* cKO mutants. Interestingly, expression of *Bmp4* was slightly decreased in the posterior SHF, but seem to be upregulated in the anterior SHF (Fig. 4I,M) as was the expression of *Isl1* (Fig. 4K,O) in *Flk1* cKO mutants. This was further corroborated by staining for ISL1 protein, which revealed accumulation of ISL1^+^ cells in the second pharyngeal arch (Fig. 4L,P). In the *Flk1* cKO mutants all the cells in the second arch and OFT were ISL1^+^, reflecting the loss of cranial NC cells, which are clearly seen in the control embryo (DAPI^+^ ISL1^−^ in Fig. 4L). In summary, our findings suggest that the incorporation of first heart field progenitors into the linear heart tube was unaffected in *Flk1* cKO mutants, while SHF progenitors failed to migrate into the OFT and right ventricle, two structures that were mostly affected in *Flk1 cKO* mutants. We suggest that deployment of pharyngeal mesoderm/SHF into the cardiac OFT is mediated by EC, directly or indirectly (Fig. 4D,H).

### Endothelial cells are required for extracellular matrix remodeling

**Fig. 5.**
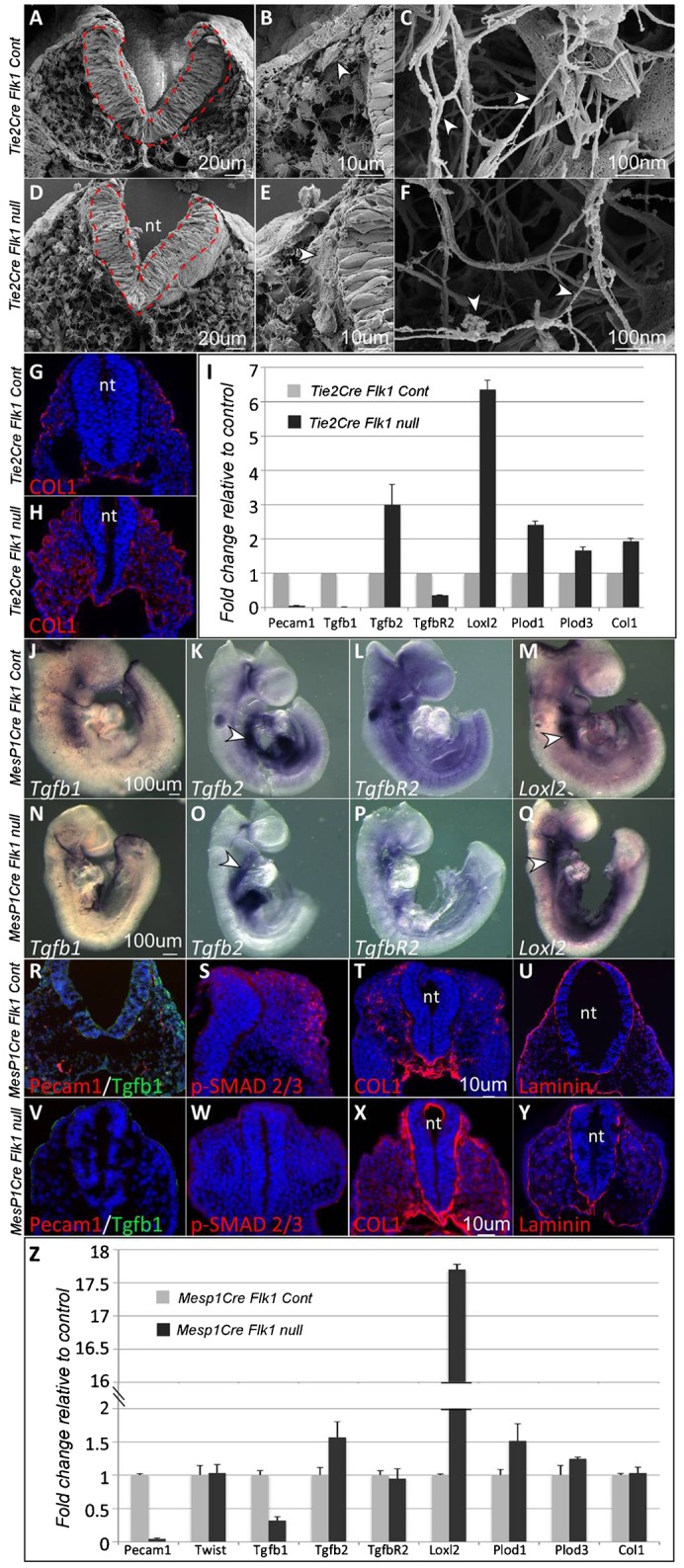
Extracellular matrix changes in *Flk1* cKO mutants. (A,D) Scanning electron microscopy of *Tie2Cre Flk1* control and mutant embryos, fractioned transversally. The neural tube is depicted in red. (B) Magnification of panel A. Arrowhead indicates a supposed NC cell. (E) Magnification of panel D. Arrowhead indicates excess ECM. (C,F) High-resolution magnification. ECM fibers morphology is different between control and mutant samples as indicated by arrowheads. (G,H) Col1 staining on sections of E9.5 *Tie2Cre Flk1* control and mutant embryos. (I) Heads of E9.5 *Tie2Cre Flk1* control (n = 2 pools of 3) and mutant (n = 2 pools of 3) embryos were isolated and analyzed for real time gene expression. (J–O) In situ hybridization in E9.5 *Mesp1Cre Flk1* control and mutant embryos for (J,N) *Tgfb1*, (K,O) *Tgfb2*, (P,L) TgfbR2 and (Q,M) Loxl2. Arrowheads indicate the region of the SHF. (R–Y) Immunostaining on transverse sections of E9.5 *Mesp1Cre Flk1* control and mutant embryos. (R,V) Co-staining for TGFb1 and PECAM1. (S,W) Immunostaining for p-Smad 2/3, (T,X) Collagen1 and (U,Y) Laminin. (Z) Heads of E9.5 *Mesp1Cre Flk1* control (n = 3 pools of 3) and mutant (n = 3 pools of 3) embryos were isolated and analyzed for real time gene expression. All fluorescent images are counterstained with DAPI (blue). Error bars indicate SD. nt, neural tube. Scale bars: 20 µm (A,D), 10 µm (B,E), 100 nm (C,F), 10 µm (G,H,R–Y), 100 µm (J–Q).

The migration of the cranial NC and SHF progenitors was perturbed in both *MesP1Cre* and *Tie2Cre Flk1* mutants, yielding severe cardiac and craniofacial defects. Furthermore, a substantial decrease in the proliferation of the neuroepithelium was detected in these mutants (Figs 1 and 2; supplementary material Fig. S1). This set of developmental processes have been previously found to be dependent on extracellular cues, we therefore hypothesized that ECM is not properly formed in *Flk1* cKO mutant embryos. Scanning Electron Microscopy (SEM) analysis of E9 embryos revealed structural differences between control and *Flk1* cKO mutant embryos. The neural tube of the mutants was thinner and deformed, validating our previous observations (Fig. 5A,D). Cells delaminating from the neural tube could be identified in the control embryo (Fig. 5B, arrowhead), but not in the mutant embryo (Fig. 5E), concurrent with the lack of NC in these mutants. An abnormal ECM was detected in the vicinity of the neural tube of the mutant embryo (Fig. 5B,E). High magnification SEM images revealed relatively straight and smooth ECM fibers in the control, compared to thinner, bent and occasionally split fibers seen in the mutants (Fig. 5C,F).

We next stained embryos' sections for Collagen 1 (COL1), a major component of the ECM (Löhler et al., 1984). COL1 was expressed abundantly and spread throughout the entire mesenchyme in the *Flk1* cKO mutants compared to the controls (Fig. 5G,H). In order to gain more insight into the molecular basis for the ECM mutant phenotype, we performed a qRT-PCR analysis on the anterior region of *Tie2Cre Flk1* mutant and control embryos (Fig. 5I). As expected from our previous findings (Fig. 1), the EC marker *Pecam1* was downregulated. Transforming growth factor beta 1 (*Tgfb1*) (ten Dijke and Arthur, 2007), implicated in both vascular and ECM development, was also decreased in the cKO mutants. *Lysyl-oxidase-like 2* (*Loxl2*), *Lysyl-hydroxilase* (*Plod*) *1* and *3*, which are collagen cross linkers that regulate ECM structure and rigidity (Kagan and Li, 2003; Myllylä et al., 2007) and *Col1* were all upregulated in *Tie2Cre Flk1* mutants compared to control (Fig. 5I).

These results were corroborated in the *MesP1Cre Flk1* mutants. In situ hybridization revealed a marked decrease in the expression of *Tgfb1* in EC in the mutant compared to control embryos (Fig. 5J,N). *Tgfb2* has a classical SHF expression pattern extending into the OFT (Fig. 5K). Its expression in the *Flk1* cKO mutants was consistent with a defect in the deployment of SHF cells into the heart as these cells remained within the distal part of the second arch (Fig. 5O). While *TgfbR2* expression pattern and levels were comparable between control and mutant embryos (Fig. 5L,P), *Loxl2* was upregulated and shifted to the anterior SHF in the *Flk1* cKO mutants compared to control (Fig. 5M,Q). Tgfb1 protein expression was downregulated in *Flk1* cKO mutants (Fig. 5R,V), as well as the phosphorylation of SMAD2/3, a readout for Tgfb signaling (Fig. 5S,W). Tgfb1 staining in the control embryo was not confined to EC, suggesting other sources of Tgfb1 in the adjacent tissues.

COL1 and Laminin staining were upregulated in *Flk1* cKO mutants, in particular in the basement membrane of the neural tube (Fig. 5T–Y). Real time gene expression analysis of both *Tie2Cre* and *MesP1Cre* mutant embryos revealed a decrease in *Pecam1* and *Tgfb1* expression relative to the control (Fig. 5I,Z). In contrast, *Tgfb2*, and the ECM modifiers *Loxl2* and *Plod1* and *Plod3* were upregulated in the *Flk1 cKO* mutants (Fig. 5I,Z). Collectively we found that the loss of EC was accompanied with a strong downregulation in both RNA and protein levels of Tgfb1, and broad changes in ECM composition and structure in *Tie2Cre* and *MesP1Cre Flk1* cKO mutants.

### Loss of Tgfb signaling in mesoderm progenitors is partially similar to second heart field phenotype observed in the Flk1 mutant embryos

**Fig. 6.**
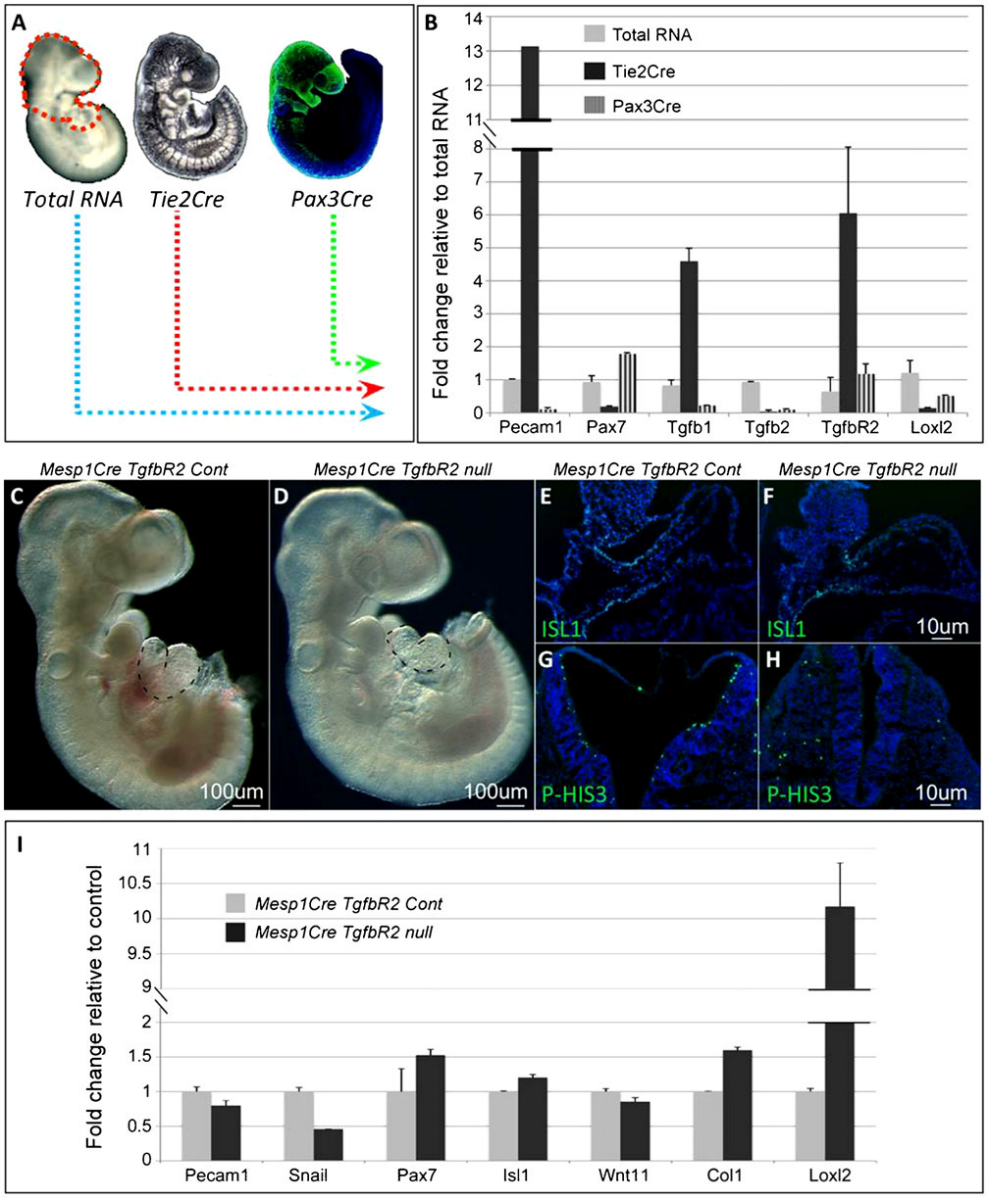
TGFb signaling at the core of the signaling circuit regulating cardio–craniofacial morphogenesis. (A) Head regions, dissected from E9.5 *Tie2Cre RYFP* and *Pax3Cre RTomato* embryos, were FACS-isolated for endothelial and NC cells, respectively. As control, the total cell population was isolated from E9.5 embryos' heads. (B) Real time gene expression in endothelial or NC cells compared to total RNA. (C–I) Analysis of *Mesp1Cre TgfbR2* E9.5 control and mutant embryos. (C,D) *Mesp1Cre TgfbR2* E9.5 control and mutant embryos. The heart is depicted by dotted lines. (E,F) ISL1 staining on sagittal sections of the OFT. (G,H) Transverse sections of the neural tube stained for the proliferation marker p-HIS3. (I) Heads of E9.5 *Mesp1Cre TgfbR2* control (n = 3) and mutant (n = 3) embryos were isolated and analyzed for real time gene expression. Scale bars: 100 µm (C,D), 10 µm (E–H).

The finding of increased *Loxl2* and decreased *Tgfb1* expression in *Flk1* cKO mutants led us to analyze the cell type specific expression of these factors and other players in the TGF beta signaling pathway. For this aim we isolated by FACS EC from *Tie2Cre Rosa26YFP* and NC progenitors from *Pax3Cre Rosa26YFP* E9.5 embryos (Fig. 6A). The enriched expression of *Pecam1* in EC (*Tie2Cre*, Fig. 6B) and *Pax7* in NC cells (*Pax3Cre*, Fig. 6B), compared to the total RNA, reflects the specificity of our FACS assay (Fig. 6A). *Tgfb1* ligand was highly enriched in EC rather than in NC cells, whereas the receptor *TgfbR2* was expressed by both cell populations.

Tissue specific ablation of TgfbR2 was previously performed in both NC and mesoderm cells (Wurdak et al., 2005; Choudhary et al., 2006; Choudhary et al., 2009). We crossed the *MesP1Cre* with the *TgfbR2* floxed allele (Fig. 6C–I). Mutant embryos appear to be normal apart from a distinct cardiac phenotype of a shortened OFT with abnormal looping (Fig. 6C,D). Sagittal sections of the embryos and ISL1 staining confirmed the shortening of the SHF and distal OFT in *MesP1Cre TgfbR2* mutants (Fig. 6E,F). In addition, we observed a reduction in the proliferation of the neural epithelium of these mutants, similar to the *Flk1* cKO mutant embryos (Fig. 6G,H). Gene expression analysis of these mutants revealed increased levels of *Col1* and *Loxl2* and a decrease in *Snail1*, a marker for migrating neural crest (Fig. 6I).

### Inhibition of VEGFR2 signaling in chick embryos recapitulates the Flk1 mutants' phenotype in the mouse

**Fig. 7.**
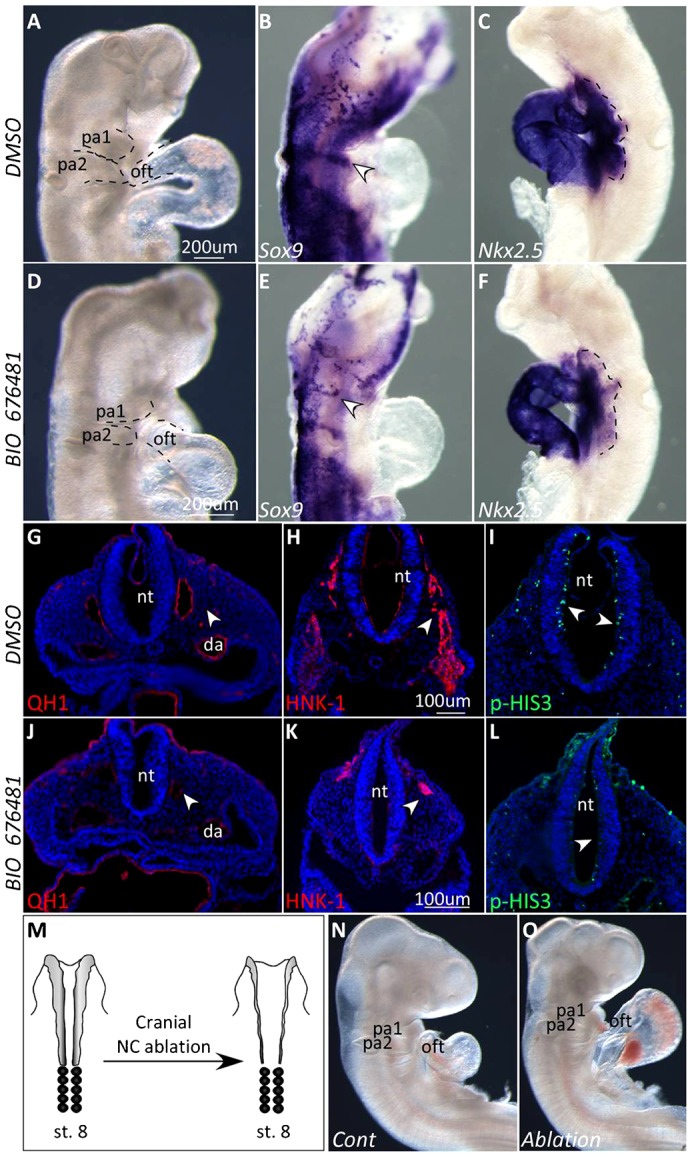
Inhibition of VEGFR2 signaling in chick embryos reveal cardiac and craniofacial defects and molecular changes resembling the mouse *Flk1* mutants. (A–L) Analysis of HH stage 13 chick embryos treated with VEGFR2 inhibitor BIO-676481 or DMSO as control. (A,D) Control DMSO-treated (n = 16/18) and BIO-676481-treated (n = 14/18) chick embryos at HH stage 13. (B,E) ISH for the NC marker *Sox9*. Arrowheads indicate streams of migrating NC cells. (C,F) ISH for the cardiac marker *Nkx2.5*. Expression in the SHF is depicted by dotted lines. (G,J) Staining for the quail endothelial specific marker QH1 in sections of control and treated embryos HH stage 13 quail embryos. (H,K) Staining for the migrating NC marker HNK-1 indicated by arrowheads. (I,L) p-HIS3 staining in transverse sections of the neural tube. Arrowheads point to differences in staining between control and treated embryos. (M) Schematic illustration of cranial neural crest ablation of a HH stage 8 chick embryo. (N,O) Stage 15 control embryo (n = 5/6) compared to a cranial NC ablated (n = 6/6) embryo. Scale bars: 200 µm (A–F), 100 µm (G–L).

In addition to the genetic ablation of Flk1 in the mouse, we used the avian model to interfere with VEGFR2 signaling using the VEGFR2 tyrosine kinase inhibitor, BIO-676481. Embryos that were treated with BIO-676481 exhibited abnormal heart morphogenesis including shortened OFT (Fig. 7A,D). In situ hybridization for *Sox9* was consistent with a fewer number of migrating cranial NC cells in BIO-treated chick embryos (Fig. 7B,E). The expression of the cardiac marker *Nkx2.5* within the heart tube was not affected while its expression decreased in the SHF of the BIO-treated embryos (Fig. 7C,F). Next, BIO-treated quail embryos were sectioned and stained for QH1, a quail specific EC marker. EC appear disorganized in BIO-treated embryos, with almost no staining in the dorsal aorta (Fig. 7G,J). The expression of the NC marker HNK1 was markedly reduced and remains confined to the dorsal region of the treated embryo, compared to the control embryo (Fig. 7H,K). We also observed a decrease in the proliferation of the neuroepithelia in BIO-treated chick embryos as shown by PH3 staining (Fig. 7I,L).

Altogether these findings show that the BIO-676486 treatment of avian embryos affected EC integrity as well as NC and SHF morphogenesis, resembling the phenotype of the *Flk1 cKO* mouse mutants. Our hypothesis was that excess ECM deposition (especially Collagen) and/or enhanced crosslinking of the collagen perturb NC migration and thus lead to the cardio–craniofacial phenotype.

It is well established that cardiac NC are important for migration of the SHF population during morphogenesis of the OFT, yet, the role of the cranial NC has not yet been addressed in this context. In the mouse, cranial NC cells from the second arch seem to enter the OFT together with ISL1^+^ cells and these cells were absent in the *Flk1 cKO* mutants (Fig. 4L,P). In order to show the importance of cranial NC in OFT morphogenesis we surgically ablated the cranial neural tube at stage 8 chick embryos. Cranial NC ablated chick embryos displayed malformations of head structures, shortened OFT and abnormal cardiac looping (Fig. 7M–O) resembling the *Flk1* cKO mouse mutants.

Taken together, our findings suggest that inhibition of VEGFR2 signaling in chick embryos affects EC integrity, NC migration, SHF looping that together result in aberrant morphogenesis of the cardio–craniofacial field. The involvement of VEGFR2 signaling and the role of EC in coordinating the cardio–craniofacial morphogenetic field are therefore conserved in vertebrates.

## DISCUSSION

**Fig. 8.**
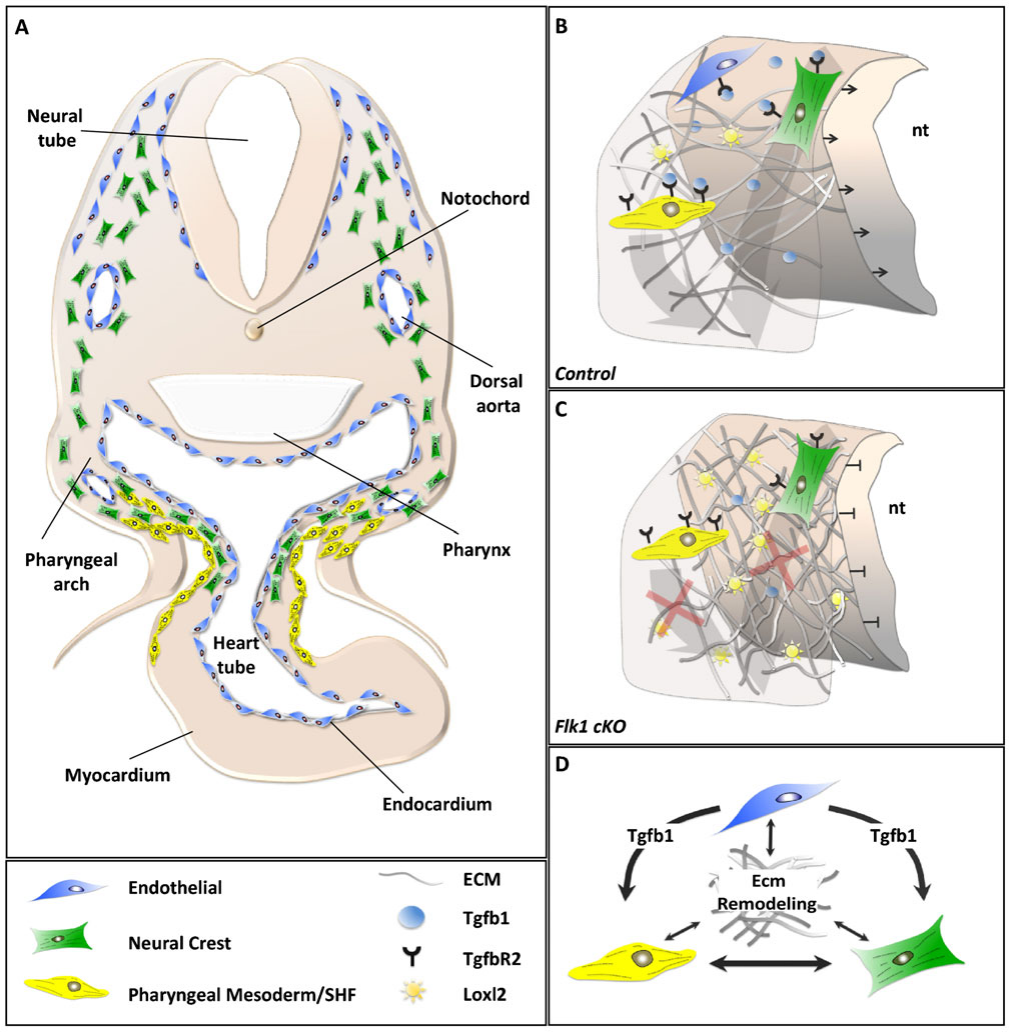
Endothelial cell signaling regulates the morphogenesis of the cardio–craniofacial field. (A) Endothelial cells (blue) are scattered throughout the entire dorso-ventral axis of the embryo, underlying the neural tube and entering the heart. Neural crest cells (green) follow the same path and enter only the most arterial pole of the OFT together with SHF progenitors (yellow). (B) The cranial progenitor niche. ECs secrete Tgfb1 that balances secretion of Loxl2 and enables proper remodeling of the ECM. (C) Loss of ECs results in reduction of Tgfb signaling and increased levels of Loxl2. Here, the ECM loses its integrity causing migratory defects of NC and SHF progenitors together with reduction in proliferation of the neuroepithelium. (D) ECM remodeling is mediated by signaling from progenitors, which in turn rely on ECM integrity for proper migration and consequent morphogenesis. nt, neural tube.

The developmental roles of EC in coordinating organogenesis are far from being clear. Several studies gradually unravel the significance of EC as organizers of early embryonic developmental processes (Cleaver and Melton, 2003). In this study, we shed light on a mechanism by which EC coordinate cardio–craniofacial morphogenesis, in part via a Tgfb1-mediated ECM remodeling program (Fig. 8). We have used conditional knockout of *Flk1* in either the anterior mesoderm (*MesP1Cre*) or more specifically in EC (*Tie2Cre*) that led to a loss of EC and abnormal development of the cardio–craniofacial field. Cranial NC migration and survival and SHF deployment into the cardiac OFT and RV were abnormal in the *Flk1* cKO mutants (Fig. 8A–C). Our data suggest that EC play a role in maintaining the integrity of the extracellular environment (Fig. 8D). Further experiments using conditional TgfbR2 knockout in mesoderm progenitors support a key role for Tgfb signaling in this developmental crosstalk. Finally, we were able to phenocopy the mouse *Flk1 cKO* phenotype in the avian model using a VEGFR2 inhibitor. We identified the collagen crosslinking enzyme LoxL2 as a candidate gene within the ECM remodeling program in the cardio–craniofacial field.

As in many studies on EC signaling, a major issue is to uncouple the signaling from the metabolic (systemic) roles of EC. It is likely that the loss of EC affects both functions. Consistent with this, Hypoxia-inducible factor-1 alpha (HIF-1a) was shown to induce *LoxL2* mRNA transcription in fibroblasts and renal tubular epithelial cells (Higgins et al., 2007), suggesting that the absence of perfusion contributes to the overall phenotype. Nevertheless, our data also suggest an EC-mediated signaling circuit. The EC-mediated cardio–craniofacial phenotype occurs as early as E8.5 when circulation is just beginning and the majority of the embryo is hypoxic (Dunwoodie, 2009). At E9.5 both control and cKO mutant embryos showed specific regions of low oxygen levels, but without significant differences between them. In addition, the molecular effects were specific for cranial NC but not pharyngeal mesoderm markers. Further, the hearts of the mutants were still beating and we were able to retrieve viable mutant embryos as late as E10.5 suggesting that the embryos at E9.5 are not dying. We could show functional EC–NC crosstalk in explant culture assays, eliminating hypoxia or absence of perfusion as major causes for the phenotype.

Tgfb1 signaling has been shown to be important for EC development as well as other cell types. Specifically, EC-derived Tgfb1 was shown to promote smooth muscle differentiation of trunk NC (Shah et al., 1996). Genetic ablation of Tgfb signaling in the NC, using *Wnt1Cre* mice, revealed a wide spectrum of craniofacial and cardiovascular defects including specific features of DGS (Ito et al., 2003; Wurdak et al., 2005; Choudhary et al., 2006). Conditional knockout of the Tgfbr2 in the mesoderm was previously performed. *MesP1Cre* mutants die at E10.5 but *Mef2c-AHFCre* mutants survive to E14 and display dilated pulmonary trunk with ruptures. The ECM was highly disorganized in the pulmonary trunk and ascending aorta (Choudhary et al., 2009). We show that *MesP1Cre TgfbR2* mutants display SHF phenotype of shortened OFT, abnormal looping and reduced ISL1 expression. Collectively these studies suggest that Tgfb signaling is required in both neural crest and pharyngeal mesoderm to control overlapping and distinct morphogenetic events within the cardio–craniofacial field. Similarly, Semaphorins and VEGF signaling molecules, acting through Npn1 (Gu et al., 2003) or PlexinD1 (Gitler et al., 2004) in EC, were shown to regulate cardiac outflow tract development. Interruption of these pathways in mice resulted in congenital heart defects as well as vascular patterning defects. Together with our findings, there is a strong evidence for a key role of EC in orchestrating critical aspects of cardiac and craniofacial morphogenesis.

DGS patients are characterized with hemizygous microdeletions of chromosome 22q11.2. The T-box containing family of transcription factors TBX1 is located within this region and haploinsufficiency of this gene promotes the manifestations of DGS in humans. A common denominator of the organs that are affected in DGS is their dependence on NC cells (Kochilas et al., 2002). However, Tbx1 is not expressed and does not function in NC cells. We have recently revealed a genetic link between Tcf21, Tbx1, and Lhx2 within the pharyngeal mesoderm. Genetic perturbation of these factors resulted in specific DGS-like phenotypes (Harel et al., 2012). Furthermore, defective vascular organization and EC dysfunction were recently shown to give rise to DGS-like phenotypes (Zhou et al., 2012).

Perturbation of EC development causes a distortion of ECM structures, which in turn affects ECM-mediated neural crest migration (Henderson and Copp, 1997; Coles et al., 2006). Defects in NC cell migration often lead to cell death (Maynard et al., 2000), as we observed in *Flk1* cKO mutant embryos. Our data suggest that one of the functions of EC is to modify the matrix in order to facilitate NC migration.

Cardiac NC cells have been shown to have a role in SHF development (Waldo et al., 2005). Furthermore, studies from our lab indicated that cranial NC cells are intimately involved in a crosstalk with pharyngeal mesoderm progenitors (including SHF cells) as they approach the distal heart tube (Tirosh-Finkel et al., 2006; Rinon et al., 2007; Tirosh-Finkel et al., 2010). Heart looping defects were evident in cranial NC ablated chick embryos (Fig. 7). Therefore, the SHF phenotype observed in both mouse and chick embryos could be attributed to the perturbed crosstalk between (cardiac and cranial) NC and SHF progenitors. Another possible explanation for this is a direct role for EC on SHF cell migration (Fig. 8A).

We propose that tissue crosstalk within the cardio–craniofacial field is extensive and critical for proper embryonic development. Our findings provide insights into some of the molecular events that are at the core of this tissues crosstalk. We suggest that EC dysfunction (by loss of VEGF signaling) results in downregulation of Tgfb1, which affects both NC- and pharyngeal mesoderm/SHF progenitors morphogenesis. ECM remodeling genes appear to be directly affected by the loss of TGFb signaling, in particularly Col1 and LoxL2. We suggest that any tissue or molecular perturbation within the cardio–craniofacial field is likely to give rise to cardiac, pharyngeal, and craniofacial defects as seen in DGS patients, due to the tight signaling circuit between all tissues.

LoxL2 is member of the Lysyl oxidase (LOX) protein family, which is made up of copper-containing enzymes that catalyze the oxidative deamination of the ε-amino groups in lysines (Smith-Mungo and Kagan, 1998). The striking upregulation of LoxL2 in *MesP1* and *Tie2 Flk1* cKO mutants as well as in the *MesP1 TgfbR2* cKO mutants (Figs 5 and 6) emerge as an important feature of the cardio–craniofacial phenotype. While we addressed the involvement of LoxL2 as an ECM remodeling modifier it is important to note that this protein had been implicated in the regulation of EMT and metastasis formation via interaction with Snail1 (Peinado et al., 2005). In addition, it was recently documented that LoxL2 is a histone modifier enzyme that catalyzes H3K4me3 deamination (Herranz et al., 2012). Thus, the roles of LoxL2 as a key regulator of the cardio–craniofacial morphogenetic field require further investigation.

## Supplementary Material

Supplementary Material
