# Supplementary material for: Endothelial cells regulate neural crest and second heart field morphogenesis
Source: Biol Open. 2014 Jul 4;3(8):679–88. doi: 10.1242/bio.20148078 (PMC4133721; doi:10.1242/bio.20148078)
Supplement: Supplementary Material [file supp_3_8_679__index.html]

Endothelial cells regulate neural crest and second heart field morphogenesis — Endothelial cells regulate neural crest and second heart field morphogenesis — Supplementary Material 

# Endothelial cells regulate neural crest and second heart field morphogenesis

## bio.20148078 Supplementary Material

**Files in this Data Supplement:**

- Supplementary Material - Michal Milgrom-Hoffman et al. doi: 10.1242/bio.20148078
