# Supplementary material for: Endothelial cells regulate neural crest and second heart field morphogenesis
Source: Biol Open. 2014 Jul 4;3(8):679–88. doi: 10.1242/bio.20148078 (PMC4133721; doi:10.1242/bio.20148078)
Supplement: Supplementary Material [file supp_bio.20148078_bio.20148078-s1.pdf]

Biology Open (2014) 000, 1–10 doi:10.1242/bio.20148078

**Supplementary Material**

**Michal Milgrom-Hoffman et al. doi: 10.1242/bio.20148078**

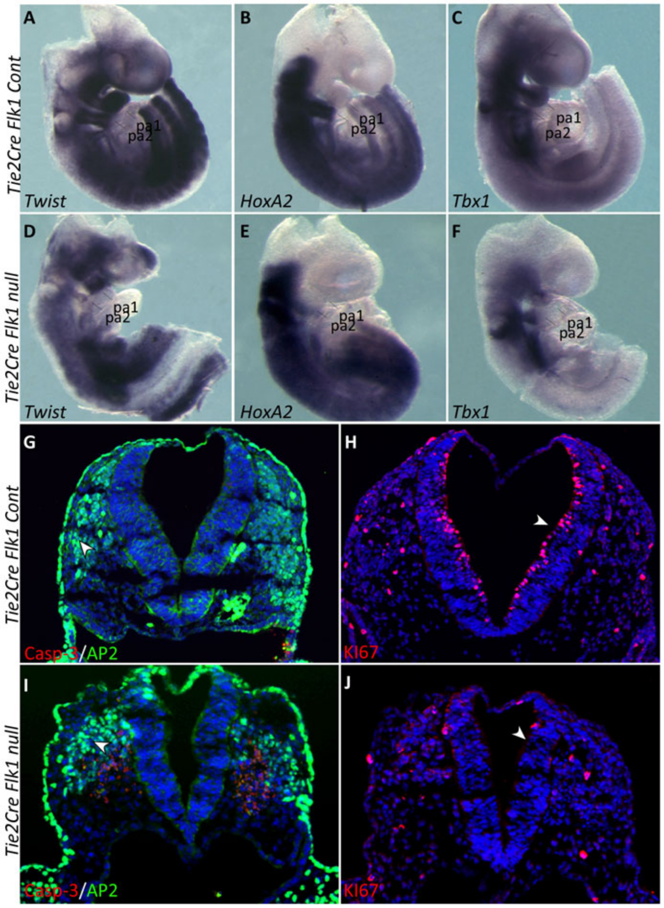


**Fig. S1. Neural crest and mesoderm gene expression in Tie2Cre Flk1 mutants.** In situ hybridization on E9.5 *Tie2Cre Flk1* control and mutant embryos for NC markers (A,D) *Twist* and (B,E) *HoxA2*. (C,F) In situ expression of the mesodermal marker *Tbx1*. (G,I) Co-staining for the NC marker AP2 and Casp-3 cell death marker. (H,J) KI67 staining of E9.5 control and mutant transverse sections at the region of the neural tube. Fluorescent images are counterstained with DAPI (blue). pa, pharyngeal arch.

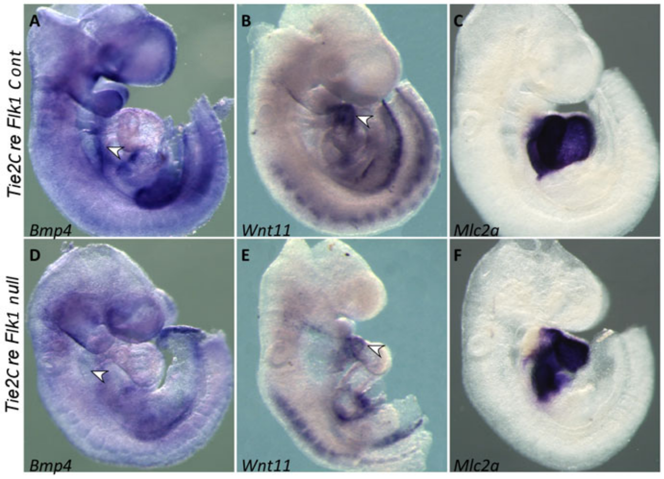


**Fig. S2. Second and first heart field gene expression in Tie2*Cre Flk1* mutants.** In situ hybridization in E9.5 *Tie2Cre Flk1* control and mutant embryos. (A,D) Expression of *Bmp4* marked by arrowheads. (B,E) In situ expression of *Wnt11* in the region of the outflow tract indicated by arrowheads. (C,F) *Mlc2a* expression marking the first heart field.

Biology Open
